# Assessment of Health Values, Beliefs, Norms, and Behavior towards Consumption Intention of 3D-Bioprinted Meat

**DOI:** 10.3390/foods13172662

**Published:** 2024-08-23

**Authors:** Mary Christy O. Mendoza, Jenn Christzel D. Chico, Ardvin Kester S. Ong, Rafael Alfredo M. Regayas

**Affiliations:** 1 School of Industrial Engineering and Engineering Management, Mapúa University, 658 Muralla St., Intramuros, Manila 1002, Philippines; 2 School of Graduate Studies, Mapúa University, 658 Muralla St., Intramuros, Manila 1002, Philippines

**Keywords:** 3D-bioprinted meat, consumer intention, health belief model, value–belief–norm theory, sustainable food alternatives

## Abstract

Continuous innovation in product development further enhances consumer appeal and contributes to a more sustainable and ethical food system. This study used the health belief model (HBM) and value–belief–norm (VBN) theory to investigate the customer perceptions of and intentions towards 3D-bioprinted meat. Specifically, this study examined consumer behavior factors using higher-order partial least squares structural equation modeling (PLS-SEM). Data were collected from 738 meat consumers through online survey questions, distributed among social groups and face-to-face distribution—limiting only to respondents who are familiar with 3D-bioprinted meats. Using a filtering question, only those who are familiar with and have knowledge of the topic were considered valid respondents. Based on the results, all variables under the integrated theories were deemed significant. Consumers’ perceptions of 3D-bioprinted meat are also shaped by altruism, egoism, biospheric concern, and willingness to change. The findings revealed that buyers rationally choose benefits over social or personal values. The study emphasized educating consumers, being transparent about production, and constantly innovating for higher acceptance of 3D-bioprinted meat. In order to foster consumer confidence, it is essential to prioritize transparency in the production process, encompassing information regarding sourcing and manufacturing methods. Certifications that validate safety and quality standards serve to reinforce this notion. In addition, the implementation of competitive pricing strategies has the potential to enhance the accessibility of 3D-bioprinted meat, whereas industry partnerships can aid in distribution operations and improve market visibility—all of which extend the practical implications developed for this study. Moreover, the foundation of the integrated framework promotes its extension and application outside technology-based meat production. This could also be considered and utilized among other studies on developed food and food consumption.

## 1. Introduction

Due to the rising global per capita food consumption and population growth, meat production was expected to double by 2020 from the baseline of 250 million tons recorded in 2003, amplifying challenges associated with breeding livestock and excessive meat consumption [1,2]. Global meat consumption per capita was 34.1 kg/year in 2014–2016, with roughly 60% red meats (pork, sheep, and beef) [3]. The consumption of red meats has been even higher from 2018 to the present. This has resulted in animal welfare, resource depletion, and environmental problems. Committed meat eaters may have a negative and significant impact on animal welfare practices, which involve minimizing stress and physical injuries among meat sources, and they desire to enhance meat quality during transport, handling, and slaughter [4,5]. Furthermore, according to a report by Compassion in World Farming [6], conventional livestock production currently exploits global resources like land, water, and fossil fuels, and by 2050, the ample resources supporting intensive animal production may become depleted. Traditional meat production is also associated with climate change, such as the impact on water availability, animal and milk production, livestock diseases, animal reproduction, and biodiversity, emphasizing the need for sustainable livestock production [7]. This includes land-use and water-use changes linked to global warming potentials [8]. An illustrative case is the Baroro River Watershed in La Union, where forest coverage has experienced a reduction in both medium-sized and large patch areas, implying a potential conversion of forest land to agricultural use [9].

It was found that, especially in Europe, food consumption accounted for 30% of total greenhouse gas emissions [2]. In certain Asian economies, total greenhouse gas emissions constitute almost 40% of total emissions [10]. In relation, industrial emissions in the Philippines totaled 12.5 million tons of CO_2_ equivalent in 2021—over 10% more than last year’s emissions [11]. With this, it could be posited that there is a growing consumer preoccupation with the correlation between dietary choices and overall well-being. This has resulted in a heightened need for products that promote better health and environmental decisions [2,12].

One of the current developments in food-related technology is 3D-bioprinted meat. This new technology was utilized by Steakholder Foods, who created the first 3D-bioprinted cultivated fish product using Umami Meats grouper cells and customized bio-inks [13]. This process ensures that the source of the meat is alive following stem cell extraction, promoting environmentally friendly meat production [14]. Moreover, there are four leading 3D-printing technologies. Selective laser, sintering, and inkjet printing use powder-form materials to create custom-shaped food products quickly without pre-processing or cutting. Extrusion printing is more popular than these three technologies due to material limitations. Extrusion printing is the most widely used 3D-printing technology in the food industry because of its simplicity, equipment structure, low cost, easy operation, and its compatibility with traditional food materials. This process involves pushing or pulling a material through a shaped opening to produce a continuous form with a specific cross-sectional profile that builds up layer by layer on the printer base [15,16].

With these technological advancements, the 3D-printing process employed in the production of cell-cultured meat can be considered for production and consumption [17]. However, with limited studies, it cannot be portrayed to be a total substitute for actual meat. This innovation has said to reduce land use and waste, as well as having lower material costs and decreased energy consumption. It has been said by Mancini and Antonioli [18] that it could have a substantial positive impact on the overall environmental sustainability of the meat industry. Furthermore, as a groundbreaking technology in the food sector, 3D-bioprinted meat emerges as a compelling solution to the environmentally detrimental and unsustainable challenges faced by the meat industry [19]. As the 3D-printing process utilizes powders or pastes to swiftly create custom-shaped food products without pre-processing or cutting, the process effectively decreases raw material waste and the risk of food contamination [1,20].

This study aimed to assess the factors influencing the adoption of 3D-bioprinted meat. Specifically, higher-order structural equation modeling (SEM) was considered in this study to evaluate the complex relationships within the conceptual framework. As indicated by Sarstedt et al. [21], higher-order SEM is beneficial for the assessment of multiple path analyses by reducing the need to individualize the different paths needed to measure a target output. Therefore, a more accurate understanding of the interconnections between various constructs of the value–belief–norm theory (VBN) and the health belief model (HBM), and their impact on consumer intentions to consume 3D-bioprinted meat, may be obtained with the current framework that is considered. Moreover, the practical implications of this study lie in informing marketing strategies, policy decisions, and industry practices related to the adoption of 3D-bioprinted meat.

## 2. Related Studies and Conceptual Framework

### 2.1. Related Studies

Researchers have been actively exploring 3D-bioprinted meat due to its innovative nature, leading to extensive investigations and studies in the field, as well as perceived solutions among related study problems. Kang et al. [22] have successfully employed tendon-gel integrated bioprinting to construct a steak-like tissue from three types of bovine cells—muscle, fat, and blood capillary cells. With a total of 72 fibers assembled to mimic the alignment found in natural meat, the developed technology shows promise for creating structured and visually authentic cultured meats. In the study of Wang [23], components of 3D bioprinting using plant-based formulations for chicken nuggets and drumsticks were assessed. It was found that 20% chicken, a 1.54 mm nozzle diameter, and a 10 mm/s printing speed mimicked softer flesh with aligned fibers. The study also stressed the role of hydrogen, disulphide, and hydrophobic interactions in printed meat analogs’ structure and fibrousness. Li et al. [24] employed 3D bioprinting with a 4% GelMA—20% silk fibroin hydrogel to construct a 3D culture system for cultured meat. The resulting structures, created with porcine skeletal muscle satellite cells, showed compact muscle fibers after 16 days, highlighting the potential of this approach for fabricating porcine skeletal muscle tissue for cultured meat.

On the other hand, according to Dick and Bhandari [25], the utilization of 3D-bioprinted meat processing holds promise for enhancing health-related behavior by enabling the creation of novel, nutritionally balanced food products tailored to address individual needs and safety concerns. The study of Caulier et al. [26] concluded that consumer acceptance of 3D-printed food, driven by customization options and a positive shift in attitudes after repeated consumption, was positive. The results emphasized an alignment with pro-environmental behavior, as well as the fact that this technology potentially offers on-demand production that can reduce food waste and environmental impact, which consumers would align with. The study of Lanz et al. [14] suggested that plant-based alternatives performed the best, while 3D-printed byproduct meat or fish alternatives performed the worst across all acceptance measures. This also underscored the significance of consumers’ perceptions of healthiness and environmental friendliness as crucial factors influencing their willingness to eat alternative products, with the conclusion emphasizing the importance of effectively communicating the health- and environmental-related benefits of 3D-printed food and cellular agriculture to promote their adoption. Therefore, the assessment of beliefs, values, and norms—especially on health-related and pro-environmental behavior—have yet to be deciphered, which is needed since the technology is currently under development and promotion has not yet been started.

### 2.2. Theories

To assess this, two theories were found to completely measure people’s perceptions. The first one, HBM, is a theory that proposes that people are most likely to take preventative action if they see a health risk as significant, feel susceptible, and see fewer costs than benefits [27]. Originating in the 1950s by a group of social psychologists, HBM is utilized commonly for understanding consumers’ proper food-handling intention and its driving force when it comes to food consumption [28,29]. The study of menu labeling in the restaurant industry, as another utility of HBM, has considered HBM to assess the factors influencing customers’ use of menu labels. It was seen that perceived threats, perceived benefits, and perceived barriers, as outlined by HBM, all positively affected customers’ engagement with menu labels [30].

On another note, the VBN, designed by Stern et al. [31], aimed to investigate the psychological and normative factors influencing individuals’ pro-environmental behavior. Studies such as that of Lind et al. [32] considered this theory of assessment of pro-environmental effects on consumer choices. It was found that values and beliefs explained 58 percent of the variance in personal norms, with both personal norms and situational factors being significant predictors of reported modality choices. Therefore, a holistic assessment of people’s health-related and environmental-related values, beliefs, norms, and behavior could be achieved by the integration of both theories. This aspect, especially among food-related studies, has not yet been covered.

### 2.3. Conceptual Framework

The conceptual framework utilized considered the integration of the HBM and VBN. Based on the related studies discussed, VBN alone utilizes variables such as values, ecological worldview, awareness of consequences, and personal norms, all affecting behavioral intentions [33]. As expressed by related studies (Section 2.2), VBN has been theorized to measure sustainability aspects of human behavior—their perception on sustainability and beliefs—in accordance with the practice of the norms. In terms of this study, it could be posited that the aim of 3D-bioprinted meat is to mitigate the environmental impact of meat consumption, as well as to provide additional meat source resources. Aligning with this study, it could be posited that limited suggestions may be developed since the focus is on the specified aspect. As a reflection of food consumption and acceptance, people may opt to consider what the majority think (social norm) [34], as well as the health implications [28,35].

To the limitation of VBN use alone, this study considered the HBM, a model that aims to evaluate the health belief of consumers. HBM commonly has constructs of perceived barriers, benefits, health motivation or concerns, and self-efficacy. Other studies include perceived susceptibility and perceived severity. However, this was not included as Gumasing et al. [36] explained that these may all be part of the HBM framework when health risks are involved. Since 3D-bioprinted meats are yet to be fully commercialized for public consumption, the benchmark of the health beliefs among consumers for accepting meat production using this technology was considered.

**Figure 1:**
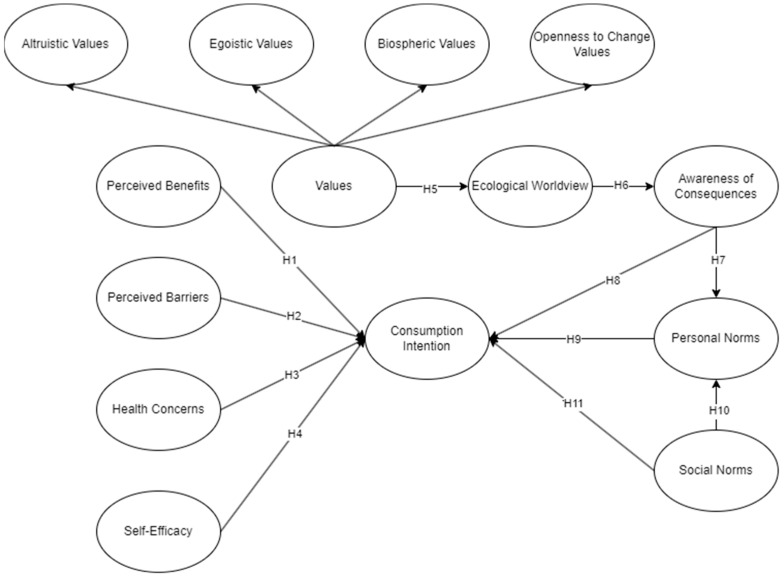
Conceptual framework.

The focus was on consumers’ attitudes, beliefs, and norms, both on aspects of health-related and pro-environmental behavior, to holistically understand the adoption of 3D-bioprinted meat. Eleven (11) hypotheses were proposed out of eleven main constructs, with higher-order variables (Figure 1).

Perceived benefits (PBNs) involve individuals’ assessment on how alternative methods mitigate disease risk, considering technology-related health risk assessments as an example [36]. On another note, de Araújo et al. [35] presented that customers consider meat quality determined by nutritional factors (i.e., fat content, sodium content, and quality), as well as environmental factors (i.e., animal welfare). A study found that customers would spend more on their preferred health and wellness food products [37]. From this study, the authors found that the primary driving factor is health consciousness, followed by considerations of product quality, flavor, packaging, and price as consumers increasingly recognize that maintaining their overall health relies on healthy food consumption—the perceived benefit among consumers [37]. Moreover, Vural et al. [38] proposed that there is a chance to encourage the acceptance of alternatives to regular meat by highlighting their perceived health benefits. Therefore, this study hypothesized the following:

**Hypothesis** **1** **(H1).** *PBNs significantly influence the intention to consume 3D-bioprinted meat.*

Perceived barriers (PBRs) refer to consumers’ concerns about adopting 3D-bioprinted meat. Meat production, including both traditional methods and slaughterhouse processes, emits greenhouse gasses, generates animal waste, consumes a lot of water, degrades the land, and contaminates poultry meat and related products with bacteria, posing severe health and economic issues for public authorities [39,40]. These are considered barriers for some consumers for their perceived consumption of different meats. In addition, health concerns (HCs) involve an individual’s sentiments regarding their overall well-being. According to Halagarda and Wojciak [41], traditional meat products may be associated with specific health safety concerns, particularly regarding their moisture content, protein levels, salt, fat, and fatty acid profile. Therefore, with 3D printing expected to solve the problems of raw material waste and food contamination, PBRs and HCs could potentially promote the acceptance of alternatives to conventional meat [1,38]. Thus, it was hypothesized as follows:

**Hypothesis** **2** **(H2).** *PBRs significantly influence the intention to consume 3D-bioprinted meat.*

**Hypothesis** **3** **(H3).** *HCs significantly influence the intention to consume 3D-bioprinted meat.*

Self-efficacy (SE) defines the belief of one’s capacity to carry out actions successfully [42]. Hidayat and Satria [43] revealed that, in the context of m-commerce, fostering self-efficacy becomes pivotal in enhancing consumption intention within the domain of m-commerce. Similarly, according to Martin et al. [44], SE plays a crucial role in the acceptance and adoption of innovative food options, like that of 3D-bioprinted meat. The study revealed that SE empowers individuals to feel confident in their ability to learn, access information, adapt, and make informed choices about innovative food options, ultimately contributing to their acceptance and adoption. Thus, this study hypothesized as follows:

**Hypothesis** **4** **(H4).** *SE significantly influences the intention to consume 3D-bioprinted meat.*

Altruistic value (AV) embodies the selfless concern for the welfare of others, emphasizing actions that benefit society without seeking personal gain [45]. A study highlighted the potential of sustainable 3D-printed meat analogs as a multifaceted solution to alleviate increasing global meat consumption, emphasizing its potential in mitigating climate change impacts and promoting responsible livestock usage [16]. On the other hand, egoistic value (EV) emphasizes evaluating actions based on their costs and benefits to one’s personal resources and interests [45]. Cabrajal-Gamboa et al. [46] revealed the potential for 3D-bioprinting technology to revolutionize the food industry by significantly improving production speed, reducing environmental pollution, and offering greater control over product attributes—ultimately benefiting manufacturers and consumers alike. With these, AV and EV can significantly influence consumer behavior, paralleling the insights from the study on 3D-printed meat in terms of environmental sustainability and consumer preferences [47].

Other values such as biospheric value (BV) prioritize protecting and preserving the natural environment for the well-being of all life [45]. Dong et al. [1] showed the opportunities of 3D-printed meat to address challenges such as raw material waste and food contamination and its opportunity to contribute valuable insights for sustainable development. A study solidifies the impact of BV on diverse sustainable behaviors, suggesting a connection between these values, individual consumption choices, and ecological worldviews [48]. Moreover, the openness to change value (OC) represents an individual’s inclination towards novelty, creativity, intellectual openness, and a willingness to explore new ideas and experiences [49]. Dong et al. [1] aligned with the broader idea of embracing and navigating through changes and innovations in the food industry, particularly in the domain of 3D printing for meat production. With this, Li and Murray [50] discovered that OC interacts positively with regard to consumer and sustainable behavior.

From these, a study of Snelgar [51] revealed that the structure of environmental concerns involves factors related to the different values. As a form of a higher-order construct to holistically assess the influence of each value [21], this study hypothesized the following:

**Hypothesis** **5** **(H5).** *Values and their higher-order constructs significantly influence ecological worldview.*

Ecological worldview (EW) measures the individual’s emotional connection with nature [52]. Hansla et al. [53] found that EW and awareness of consequence are linked by engagement with environmental issues and pro-environmental behavior, influenced by AV, EV, and BV. These are then said to be empirically linked to awareness-of-consequences beliefs and power, benevolence, and universalism. A study also found that consumer attitudes toward sustainable meat alternatives (i.e., cultured meats) are influenced by carbon emissions awareness and ethical concerns. It was highlighted that there is a connection to the broader EW and emphasizing the need to consider individual values when assessing the acceptance of alternative food and technology development for environmental sustainability [54]. Thus, it was hypothesized as follows:

**Hypothesis** **6** **(H6).** *EW has a significant influence on awareness of consequences.*

Awareness of consequences (ACs) involves recognizing the refrains from pro-environmental actions that may result in negative outcomes for others or the environment in general [45]. The study of Dong et al. [1] revealed the challenges and opportunities of implementing 3D printing of meat highlighting the importance of ACs, to which they emphasized the need to address issues such as rheological properties, complex meat colloidal systems, and limitations in printer functionality for sustainable and responsible consumption. With this, a study provided support for the notion that ACs influences consumers’ personal norms [55]. In addition, attitudes toward environmental friendliness and healthiness are important indicators of consumption intention and adoption of 3D food printing [14]. Thus, the following were hypothesized:

**Hypothesis** **7** **(H7).** *ACs has a significant influence on consumer’s PNs.*

**Hypothesis** **8** **(H8).** *ACs significantly influence the intention to consume 3D-bioprinted meat.*

Moreover, personal norms (PNs) provide an internal guide on how to behave ethically [56]. The study of Ross et al. [57] also provided insights into how individual attitudes and beliefs, including perceived personal relevance, neophobia, trust in science, and concerns about naturalness, influenced consumers’ willingness to adopt 3D-printed food applications. Similar notions in the Philippine setting can be seen in the study of Tacardon et al. [58]. Moreover, the study of Joanes [59] findings underscore the significant influence of psychological factors, particularly environmental concerns and the incorporation of IWAH, in shaping personal norms, thereby elucidating their impact on individuals’ intentions to reduce clothing consumption. Therefore, the strength of PNs could significantly influence consumer intentions, shaping their willingness to adopt novel technologies or innovations [57]. Thus, the following was hypothesized:

**Hypothesis** **9** **(H9).** *PNs have a significant influence on the intention to consume 3D-bioprinted meat.*

Furthermore, social norms (SNs), in this study, refer to the shared expectations, rules, and behaviors within a group or society that greatly influence individuals based on normative influences and the contextual aspects of others’ daily lives [34]. One study showed that values and their constructs influenced PNs for purchasing pro-environmental products, and that SNs—whether directly or indirectly—influenced pro-environmental behavior through PNs. On the other hand, Kim and Park [60] revealed that social influences can impact consumers’ decisions and adoption behavior. This is inherently connected to consumer intentions in the context of adopting innovative products. Moreover, the study of Kulviwat et al. [61] found that social influence and adoption attitude significantly affect customers’ inclination to adopt high-tech innovations. It was also indicated that a consumer’s attitude fully mediates the effect of social influence on adoption intention, stressing the relevance of individual attitudes. The study of Jia et al. [62] also underscores the mechanisms through which social norms influence personal norms and subsequent intentions to consume, drawing upon theoretical frameworks such as the Theory of Planned Behavior, Norm Activation Theory, Attribution Theory, the Economic Man Hypothesis, and Social Identity Theory. In line with this, this study hypothesized the following:

**Hypothesis** **10** **(H10).** *SNs have significant influences on PNs.*

**Hypothesis** **11** **(H11).** *SNs have significant influences on the intention to consume 3D-bioprinted meat.*

## 3. Methodology

### 3.1. Participants

The data for this study were gathered through an online survey conducted using Google Forms, which was widely distributed across various internet and social platforms, as well as face-to-face distribution among older generations. Specifically, the data collection targeted meat consumers who are familiar with 3D-bioprinted meats. Since this was advertised online, only collected respondents who have viewed and understood 3D-bioprinted meat were considered valid respondents. The filtering question in line with this was “Have you seen this new meat technology, and are you familiar with it?”. Among the 800 collected data, only 738 were considered valid—those who answered yes. Employing a stratified random sample approach, separation of meat eaters into distinct subpopulations to ensure representation from various fields was employed [63]. This survey strategy was necessary to gather complex perspectives about 3D-bioprinted meat consumption intentions.

### 3.2. Measure Items

The questionnaire employed in this study draws on a comprehensive range of investigations to assess 13 latent variables related to consumers’ attitudes and beliefs toward 3D-bioprinted meat. Derived from a total of 67 constructs adapted from related studies, the indicators were carefully chosen to capture the nuances of consumer perspectives. The questionnaire is structured to measure consumption intention (CI), altruistic value (AV), egoistic value (EV), biospheric value (BV), openness to change value (OC), ecological worldview (EW), awareness of consequences (ACs), personal norms (PNs), social norms (SNs), perceived benefits (PBNs), perceived barriers (PBRs), health concerns (HCs), and self-efficacy (SE). Adapted items from various authors, such as Jakovcevic and Steg [47], Mayer and Frantz [54], Sadeli et al. [64], Gumasing et al. [39], and Yuen et al. [65,66] contributed to the questionnaire’s robustness. Each construct comprises multiple items (Table 1), with respondents providing their responses on a five-point Likert scale, ranging from 1 (strongly disagree) to 5 (strongly agree). Prior to assessment, the questionnaire underwent pre-testing for checking coherence, grammar, and understanding, as well as psychometric validity. An acceptable output with an overall Cronbach’s alpha of 0.893 was obtained and was deemed acceptable for deployment [67].

### 3.3. Higher-Order Structural Equation Modeling

The study proposed a model that utilized reflective–reflective higher-order components. Higher-order structural equation modeling (SEM) is commonly applied to analyze causal relationships among latent variables, encompassing both traditional constructs and higher-order factors [28,67]. In this approach, higher-order constructs serve to encapsulate abstract, overarching dimensions along with their more concrete subdimension [2]. In a German study [68], it was found that there are significant correlations between perceived environmental concern, service quality, and customer satisfaction—revealing insights into factors influencing consumer behavior intentions. Their higher-order construct provided significant measures on the domains of service quality, better than lower-ordered constructs. The study of Huang [69] also provided evidence on the higher-ordered construct of technology acceptance. It is evident from these studies that utilizing higher-order SEM could help gain better insights into different domains, presenting the significant levels of each variable effectively. Therefore, the higher-order SEM approach was chosen for this study to comprehensively analyze the measured items of four value domains and eight other main constructs related to consumers’ attitudes and beliefs toward the intention to consume 3D-bioprinted meat. The utilization of SMART-PLS v3.0 was considered for the analysis in this study.

## 4. Results

### 4.1. Demographic Profile

The demographic data were collected from December 2023 to July 2024, and the descriptive statistics are shown in Table 2. Prior to responding to the survey questionnaire, the participants completed a consent form mandated by Republic Act No. 10173 (Data Privacy Act of the Philippines). The table below indicates that the age group with the highest number of respondents was in the 21–30-years-old category, constituting 32.66% of the total. Subsequently, the 41–50-years-old group comprised 26.02% of the respondents, while the 31–40-years-old group represented 24.53% and the 18–20-years-old category represented 12.87%. The age group with the fewest respondents was the 51 years old and above category, constituting 3.93% of the total. The table shows that 51.49% of the participants were female, while 48.51% were male.

The distribution of educational backgrounds among participants revealed a diverse profile. A significant portion of respondents reported completing college, 49.46%, closely followed by high school graduates, 48.78%. Elementary graduates comprised 0.95% of respondents, and a smaller proportion without any formal schooling constituted a minor percentage, 1.2%. Moreover, most participants identified themselves as employed or self-employed, comprising 50.95% of the total respondents. Students constituted the second-largest group, making up 37.53%, highlighting the active participation of the employed or self-employed demographic. Unemployed individuals represented 9.21%, and retired participants accounted for 2.30% of the sample. Moreover, respondents displayed diverse patterns of traditional meat consumption. Specifically, 33.47% opted for daily meat consumption, while 31.98% reported eating meat 3–4 times a week. A significant number of respondents, 23.04%, consume meat 5–6 times a week, and 11.11% reported a frequency of 1–2 times a week. A smaller percentage, 0.41%, abstain from traditional meat intake altogether. Additionally, participants were surveyed about their inclination toward consuming technology-produced meat.

### 4.2. Results of SEM

**Figure 2:**
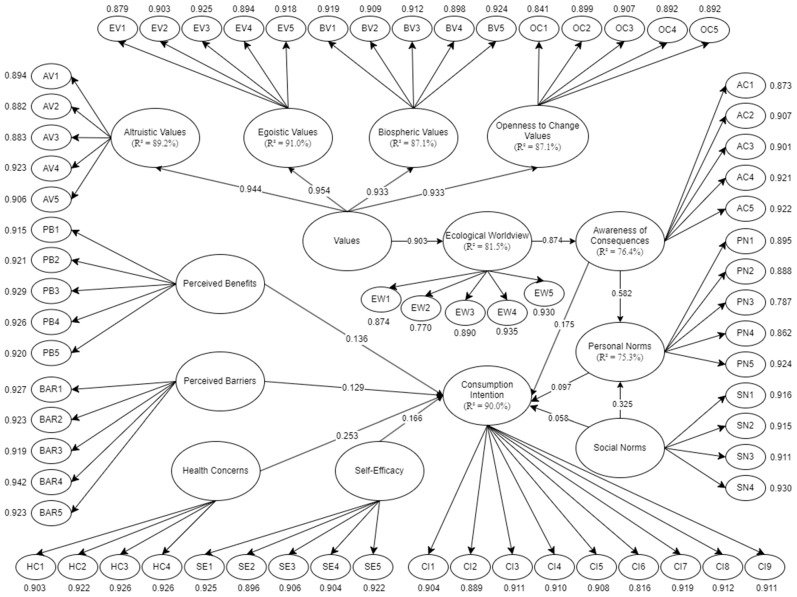
SEM for consumers’ consumption intention of 3D-bioprinted meat.

The SEM approach employed for evaluating consumers’ intention regarding 3D-bioprinted meat is illustrated in Figure 2. This model serves to validate the relationship between the observed data and constructs, with each latent variable’s indicators functioning as measuring factors. As expressed by Hair and Alamer [70], relationships will be deemed significant when the *p*-value is less than or equal to 0.05, while factor loadings should be greater than or equal to 0.70. Moreover, through computation of beta coefficients and R^2^ values, the model’s performance was evaluated. The model achieved an R^2^ value of 90.00% for consumption intention, indicating a substantial explanatory power regarding consumers’ intent to adopt 3D-bioprinted meat.

Among the influencing factors, egoistic values demonstrated the strongest impact at 91.0%, closely followed by altruistic values at 89.2%. Biospheric values and openness to change also contributed significantly, explaining 87.1% of the variance. Ecological worldview exhibited moderate explanatory power, with R^2^ values of 81.5%. Although still substantial, awareness of consequences accounted for 76.4% of the variance, and personal norms showed lower explanatory power, with R^2^ values of 75.3%. As recommended by Henseler et al. [71], an R^2^ score of 20% or higher is deemed acceptable for behavioral studies. These findings underscore the multifaceted nature of consumers’ decision-making processes, highlighting the significant roles played by both individual values and broader environmental concerns in shaping intentions towards 3D-bioprinted meat consumption.

Reflecting on the model, R^2^ values implicate a variation and contribution of the variables affecting consumption intention. Personal norms in this case represent 69.1% of the variance in terms of the consumption intention. In accordance with this, more people are aware of the environmental impacts of meat consumption, with a variance of 73.2% and 78.9% on awareness of consequences and ecological worldview, respectively. As a highlight, consumption intention spreads to 87.3% of the total variance. This means that the analysis promotes the general predictors of consumers’ intention of 3D-bioprinted meat consumption. As expressed by Byrne [72] and Amazhanova and Huseynov [73], an R^2^ value between 0.4 and 0.9 is deemed acceptable for consumer behavior studies.

The convergent reliability and validity of the final model are presented in Table 3. The mean represents the average value of a variable, while the standard deviation (S.D.) measures the variability or dispersion of the data points around the mean. Additionally, a test for data collection normality using the Shapiro–Wilk Test (SWT) yielded a value within the ±1.96 threshold, indicating a normal dataset [74]. Factor loadings (FLs) of 0.7 and above indicate successful capture of latent variable variability. Internal consistency, reliability, and validity were evaluated using Cronbach’s alpha (α) and composite reliability (CR), with a threshold of ≥0.7, and average variance extracted (AVE) with a threshold of ≥0.5 [67]. As evidenced, all metrics surpassed the required thresholds, affirming strong internal consistency, reliability, and validity across all constructs.

The discriminant validity of the structural model was assessed by applying the Fornell–Larcker criteria (FLC) and the Heterotrait–Monotrait correlation ratio (HTMT), presented in Table 4 and Table 5, respectively. This is to show a significant correlation between each latent variable and to evaluate the structural model. The results demonstrate discriminant validity since all values meet the specified thresholds [71]. The FLC technique, a conservative approach described by Hair [67], evaluates the square root of AVE for each latent variable (diagonal values). It was indicated that these must be greater than the values in the respective rows and columns for discriminant validity to be achieved. In contrast, HTMT utilizes a correlation approach based on Monte Carlo simulation, with Kline [75] suggesting a cutoff level of 0.850 to maintain discriminant validity and avoid overlap for separate constructs.

### 4.3. Model Fit Analysis

The reliability of the proposed model is demonstrated in Table 6. The results suggested that all parameter estimates surpassed the minimum threshold value as suggested, thereby confirming the appropriateness of the proposed model. Furthermore, bootstrap samples are also drawn from the modified data. This modification involves utilizing the model-implied correlation matrix after orthogonalizing or standardizing all variables. Henseler and Djisktra [76] suggested that if more than 5% of bootstrap samples have discrepancy values greater than the actual model, the sample data may come from a population that acts as expected.

The PLS-SEM was conducted to examine the given hypotheses for the SEM, presented in Table 7. It could be seen that, under HBM theory, perceived benefits (β = 0.136, *p* = 0.005), perceived barriers (β = 0.129, *p* = 0.009), health concerns (β = 0.253, *p* = 0), and self-efficacy (β = 0.166, *p* = 0) have positive and significant effects on consumption intentions of 3D-bioprinted meat. Moreover, under the higher-order construct, values have a positive and significant influence on egoistic value (β = 0.954, *p* = 0), altruistic value (β = 0.944, *p* = 0), openness to change value (β = 0.933, *p* = 0), and biospheric value (β = 0.933, *p* = 0). It can also be seen that values affect ecological worldview (β = 0.903, *p* = 0), which affects awareness to consequences (β = 0.874, *p* = 0), then affects personal norms (β = 0.582, *p* = 0) and consumption intention to 3D bioprinted (β = 0.175, *p* = 0). Furthermore, personal norms affect consumption intention (β = 0.097, *p* = 0). Additionally, social norms influence personal norms (β = 0.325, *p* = 0) and consumption intention (β = 0.058, *p* = 0.047).

## 5. Discussion

This study examined consumers’ health-related and pro-environmental attitudes, values, beliefs, and norms to understand 3D-bioprinted meat consumption. The factors affecting 3D-bioprinted meat consumption intention were determined using PLS-SEM, which examined eleven constructions. The results indicate that values and their higher-order constructs have the most decisive, significant, and positive influence on ecological worldview (β = 0.903, *p* < 0.001). The reflective-ordered construct results suggest that individuals’ values, particularly those related to EV (β = 0.954, *p* < 0.001), AV (β = 0.944, *p* < 0.001), OC (β = 0.933, *p* < 0.001), and then BV (β = 0.933, *p* < 0.001), significantly influence their perception of 3D-bioprinted meat. These findings highlight the importance of considering diverse value orientations in shaping consumer attitudes and behaviors towards emerging technologies in food production, which is supported by the study of Anders et al. [80]. This study could therefore contribute to understanding the complex interplay between values and ecological worldviews, providing insights into the factors influencing acceptance and adoption of sustainable food alternatives like 3D-bioprinted meat.

The correlation between EV and EW implied that individuals who are concerned about personal gains and resources may also perceive 3D-bioprinted meat favorably, considering its potential to offer sustainable food options without compromising personal interests [45,46]. Individuals may view this alternative as a means to enjoy meat consumption while minimizing harm to animals and the environment, thereby fitting within their ethical values and beliefs. This also highlights personal benefits and advantages of consuming 3D-bioprinted meat, such as sustainability and ethical considerations, which may resonate more strongly with individuals who prioritize their own interests [81].

On the other hand, AV on EW suggests that individuals who prioritize altruistic values are more inclined to view 3D-bioprinted meat as a solution benefiting both society and the environment. This alignment is consistent with its potential to mitigate climate change impacts and encourage responsible consumption. Furthermore, this relationship sheds light on how individual values shape attitudes toward 3D-bioprinted meat, indicating that people perceive AVs as a way to contribute to societal well-being and environmental sustainability in line with their altruistic beliefs [82].

Moreover, the correlation between OC and EW demonstrates that individuals with a tendency for embracing novelty and change are inclined to see 3D-bioprinted meat as an exciting and innovative solution to food production, aligning with their openness to exploring new ideas and experiences [51,52]. It suggests that individuals with a greater propensity for embracing novelty perceive 3D-bioprinted meat as an exciting and innovative option in the realm of food production. Additionally, this inclination toward openness to change may indicate a heightened environmental consciousness among individuals, leading them to be more cognizant of the ecological implications of their consumption choices [83]. As a result, promoting openness to change could serve as a strategy for fostering greater acceptance and adoption of sustainable food alternatives like 3D-bioprinted meat.

In addition, the positive relationship between BV and EW suggests that those who prioritize environmental preservation are likely to view 3D-bioprinted meat as a favorable option due to its potential to reduce the ecological footprint associated with traditional meat production. Reflecting on related studies with the same connotation, it could be posited that this contributes to the well-being of the planet. This implies that emphasizing the environmental benefits of 3D-bioprinted meat, such as reducing carbon emissions, minimizing land and water usage, and decreasing pollution associated with traditional meat production, could resonate strongly with consumers who prioritize environmental preservation.

The second interesting finding underscores a positive and significant impact of EW to AC (β = 0.874, *p* < 0.001). This implies that those who view 3D-bioprinted meat as a way to protect the environment, honor animal lives, and promote environmental health are more likely to understand its benefits and drawbacks. Values and emotional connection to nature could be said to strongly influence people’s awareness of the broader consequences of their behavior, especially when adopting breakthrough food technology like 3D-bioprinted meat. This assertion resonates with the observations of Pakseresht et al. [54], who emphasized the significance of considering individual values and emotional connections to nature in evaluating attitudes towards alternative food technologies for environmental sustainability.

The third interesting result of the study is the positive relationship of AC and consumers’ PNs (β = 0.582, *p* < 0.001), indicating that individuals who are more aware of the potential consequences of their actions regarding 3D-bioprinted meat tend to have stronger personal norms related to its consumption. It could be posited that individuals who understand the potential benefits of 3D-bioprinted meat, such as reducing environmental impact and promoting sustainability, are more likely to integrate ethical considerations into their PNs [1,55].

The study also found a positive influence of SNs to PNs (β = 0.325, *p* < 0.001). This suggests that individuals who perceive social pressure or influence from their peers and social networks regarding the consumption of 3D-bioprinted meat are more likely to intergrade these norms into their own personal beliefs and behaviors. Interventions aimed at promoting sustainable consumption practices should consider leveraging SNs to influence individuals’ attitudes and behaviors positively [84]. By creating a supportive environment and promoting sustainable consumption, stakeholders may stimulate the adoption of environmentally friendly alternatives like 3D-bioprinted meat [85].

The fifth interesting finding underscores the significant impact of HCs (β = 0.253, *p* < 0.001) to the intention of consuming 3D-bioprinted meat. This implies that individuals with HCs, including worries about unknown diseases and the safety of conventional meat, are more likely to perceive 3D-bioprinted meat as a safer and healthier option, which is supported by the studies of Godoi et al. [86] and Portanguen et al. [87]. The belief that 3D-bioprinted meat can safeguard both health and financial stability further underscore its potential as a viable alternative to traditional meat products. However, it is worth noting that studies still indicate consumer uncertainties regarding the safety of 3D-bioprinted meat, which could impact consumer acceptance [14,44,54].

Furthermore, AC significantly influences the intention to consume 3D-bioprinted meat (β = 0.175, *p* < 0.001). This suggests that individuals who are more aware of the potential positive outcomes and benefits associated with consuming 3D-bioprinted meat are more likely to express an intention to consume it. Specifically, individuals who believe that 3D-bioprinted meat has the potential to reduce reliance on finite resources, improve quality of life, contribute to a cleaner environment, and promote respiratory health are more inclined to express an intention to consume it. Additionally, those who perceive no negative consequences in consuming 3D-bioprinted meat, believe in its safety for consumption, and see potential societal benefits from its development and consumption are also more likely to express an intention to consume it. This implies that individuals’ awareness of the positive consequences and benefits associated with 3D-bioprinted meat plays a significant role in shaping their intention to consume it [88,89].

This study also revealed that SE significantly influences the intention to consume 3D-bioprinted meat, showing a positive relationship (β = 0.166, *p* < 0.001). The study shows how SE helps people to learn, obtain information, adapt, and make educated decisions about innovative food options, facilitating their acceptance and adoption for consumption. This shows that people with stronger SE are more likely to prefer and embrace this innovative food alternative [90]—fitting with SE as the belief in one’s ability to succeed [42]. According to Martin et al. [44], SE is crucial to the adoption of new foods like 3D-bioprinted meat.

Moreover, the study found that PB, encompassing health consciousness, safety, sustainability, and ethical concerns, significantly influences consumers’ intentions to consume 3D-bioprinted meat (β = 0.136, *p* = 0.005). This suggests that individuals who recognize 3D-bioprinted meat’s potential to transform the food industry, reduce reliance on animal agriculture, and address environmental concerns are more inclined to consider it. On a related note, health and safety concerns are also said to be paramount due to the risks associated with conventional meat production [35,54]. Additionally, growing environmental awareness fosters support for sustainable food systems, aligning with 3D-bioprinted meat’s perceived sustainability [16]. Ethical considerations regarding animal welfare also play a significant role, with 3D-bioprinted meat offering a more humane alternative, thus influencing consumption intentions [1]. Therefore, consumers’ intentions to consume 3D-bioprinted meat are significantly influenced by health, safety, sustainability, and ethical considerations, indicating its potential to transform the food industry while addressing environmental and ethical concerns.

The ninth interesting finding underscores the significant impact of BAR (β = 0.129, *p* = 0.009) on the consumption intention. Consumers who perceive limitations in traditional meat production, such as contamination hazards, environmental impact, and animal suffering, are more inclined to explore 3D-bioprinted meat, which is similar to the implications of Verbeke et al. [91]. This observation is consistent with prior studies which have also highlighted the potential of 3D-bioprinted meat in addressing these concerns.

Finally, this study found that PNs had a significant impact on the intention to consume 3D-bioprinted meat (β = 0.097, *p* < 0.001), and SNs exhibited a significant positive effect (β = 0.058, *p* = 0.047). These results suggest that individuals’ intentions to adopt 3D-bioprinted meat are markedly influenced by either perceived social pressure or internalized personal convictions pertaining to environmental and ethical considerations. The study of de Groot et al. [56] found that the efficacy of normative messages in influencing pro-environmental behavior is influenced by individual personal norms, with individuals possessing stronger personal norms demonstrating increased engagement in environmentally conscious food and dietary behaviors regardless of societal norms. This is supported by a study that revealed that individuals with stronger personal norms regarding 3D-bioprinted meat are more likely to express an intention to consume it [57].

The study revealed that social norms significantly influence consumption intentions for 3D-bioprinted meat due to its novelty and limited engagement in conversations surrounding it. With this, the findings have underscored the role of social effects in consumer choice, particularly for innovative and ecological products. The study of Lanz et al. [14] suggests that there should be a shift in attention towards promoting the health and environmental advantages of 3D food printing and cellular agriculture to encourage their uptake in the future. Moreover, the study aligns with previous research by Kim and Seock [34], Kim and Park [60], and Kulviwat et al. [61], indicating the interconnectedness of SNs, PNs, and consumer behavior. Thus, by emphasizing the environmental and ethical benefits of 3D-bioprinted meat, policymakers, marketers, and advocates can potentially influence individuals’ personal norms, thereby increasing the likelihood of widespread acceptance and consumption of this alternative protein source [54,92].

### 5.1. Practical Implication

The study’s practical implications provide stakeholders engaged in promoting and facilitating the adoption of 3D-bioprinted meat with actionable guidance. It is crucial that educational campaigns prioritize the perceived advantages of 3D-bioprinted meat, encompassing its capacity to mitigate health concerns, foster ecological sustainability, and adhere to ethical standards. By utilizing the findings regarding the impact of values and beliefs on consumer attitudes, individual advertisements have the ability to resonate with various value orientations. Establishing these distinctive selling factors at the forefront of product positioning strategies should demonstrate the health, environmental, and ethical benefits of 3D-bioprinted meat. Therefore, management is advised to consider the transparency of production processes and health protocols. By allowing for proper health and safety certification, intention for 3D-bioprinted meat consumption may be heightened among consumers.

To further invoke consumer confidence, it is essential to provide information regarding sourcing and manufacturing methods. Validated safety and quality standards serve to reinforce this notion and could be highlighted when promoting 3D-bioprinted meat for consumption. The implementation of competitive pricing strategies has the potential to enhance the accessibility of 3D-bioprinted meat, whereas industry partnerships can aid in distribution operations and improve market visibility. It is suggested that companies associate with established brands to further the commercialization of 3D-bioprinted meats, with trust being easily obtained among partnered brands. It is also critical to gain consumers via social media platforms, organize educational seminars, and organize events in order to cultivate awareness and establish further trust. By offering consumers the chance to engage with the product, inquire about its advantages, and pose concerns, it is possible to clarify misunderstandings and stimulate trial usage.

Moreover, ensuring consumer confidence and safety is reliant upon regulatory compliance, whereas continuous pursuit of innovation in product development enhances consumer appeal. Contributing to a more sustainable and ethical food system, stakeholders and management can advance the acceptability and adoption of 3D-bioprinted meat by formulating targeted strategies informed by the findings of this study.

### 5.2. Theoretical Implication

This study integrated the HBM and VBN theories to provide a complete theoretical framework for analyzing consumers’ attitudes and intentions regarding the consumption of 3D-bioprinted meat. This study offers a detailed understanding of the factors that influence consumer behavior in the context of developing food technologies and also incorporates key components from both theories. However, the HBM alone may have limitations in capturing the broader societal and normative influences on consumer behavior beyond health-related perceptions, potentially overlooking factors such as environmental concerns and ethical considerations [34]. Similarly, while the VBN theory provides insights into the psychological and normative factors influencing individuals’ choices, it may not fully account for health-related perceptions and the specific risk–benefit evaluations characteristic of the HBM [37]. By integrating both theories, the model addressed these limitations, allowing for a more nuanced understanding of consumer behavior toward 3D-bioprinted meat. The integrated approach enables researchers and practitioners to consider a broader range of factors, including health beliefs, societal values, and environmental concerns, when developing strategies to promote the acceptance and adoption of novel food technologies.

As suggested by Hair et al. [67] and Sarstedt et al. [21], utilizing higher-order SEM enriches the study by enabling the investigation of complex relationships among various components—which has not been considered for VBN and health-related studies. This study enhances the present state of knowledge by clarifying how health-related beliefs and broader societal values influence attitudes towards consuming 3D-bioprinted meat. As a result, it provides valuable insights for future research to consider in the areas of consumer behavior and acceptance of food technology. Moreover, the framework utilized could be used in different areas of study, even covering diverse domains such as energy consumption, sustainability practices, and environmental conservation efforts, where understanding the interplay of individual beliefs, societal norms, and values is crucial for informing policy and behavior change initiatives.

### 5.3. Limitations and Suggestions for Future Research

Despite the contributions made by this study, several limitations should be acknowledged. Firstly, the data collection method relied on a stratified random approach. Despite being subjectively appropriate among consumer behavior and human factors studies [93], future research could employ more diverse sampling techniques to ensure a broader representation of the population, and employing a qualitative approach may provide more insights into the consumption intention and decipher other significant factors. This study was only able to collect information from individuals of younger generations who are familiar with the production of 3D-bioprinted meat. Despite significant findings, it is believed that different age groups would have different perceptions on acceptance, technology adoption, and sustainability. Since this study was able to collect only from individuals familiar with 3D-bioprinted meat, future research may conduct interviews to provide insights from those who are not familiar with the technology and consider this study as benchmark for the acceptance of 3D-bioprinted meats in the Philippines among younger generations.

Second, the study focused primarily on consumer attitudes and intentions toward 3D-bioprinted meat, overlooking other potential factors such as cultural influences and economic considerations since an establishment of the model was considered. Subsequent research could explore other factors to provide a more comprehensive understanding of consumer behavior in this domain; some examples of which may be the utility of the theory of planned behavior for overall behavioral analysis, protection motivation theory—for any protective behavior assessment—and even technology acceptance models. Additionally, while this theoretical framework integrated the HBM and VBN theories, other theoretical perspectives could be incorporated to enrich the analysis further. Finally, the study utilized self-reported measures, which are subject to social desirability bias and may not always reflect actual behavior, with limitations in terms of theory-based analyses. Future research could employ experimental designs or observational studies—and even face-to-face interviews—to validate the findings and provide a more robust assessment of consumer behavior, or conduct interviews to obtain substantial insights for a qualitative–quantitative approach.

## 6. Conclusions

This study used the integrated HBM and VBN theories to investigate the variables influencing consumers’ intention to consume 3D-bioprinted meat. A questionnaire was generated and distributed to a total of 738 meat consumers using stratified random sampling. The study employed higher-order partial least squares structural equation modeling (PLS-SEM) to simultaneously evaluate and validate consumer attitudes, beliefs, and norms related to health and pro-environmental behavior. It was revealed that significant correlations between perceived barriers, perceived benefits, self-efficacy, and health concerns substantially impacted consumers’ intentions. Additionally, higher-order constructs of values such as altruism, egoism, biospheric concern, and openness to change significantly shaped consumer attitudes, subsequently influencing their consumption intentions. Interestingly, social norms and personal norms did affect the intention to consume 3D-bioprinted meat, indicating a more rational decision-making process based on perceived benefits. These insights provide a foundation for informed decision-making and targeted interventions to encourage the acceptance and adoption of 3D-bioprinted meat. Stakeholders should prioritize consumer education on the health, environmental, and ethical advantages of 3D-bioprinted meat.

## Figures and Tables

**Table 1:** Measured items.

Construct	Items	Measures	References
Consumption Intention	CI1	I have the intention to consume 3D-bioprinted meat because I believe it would create a revolutionized and sustainable meat substitute.	[36,45,52,64,65,66]
	CI2	I have the intention to consume 3D-bioprinted meat because of ethical reasons.	
	CI3	I have the intention to consume 3D-bioprinted meat to reduce animal agriculture needs.	
	CI4	I would consider 3D-bioprinted meat because of the current technology development.	
	CI5	I would consider 3D-bioprinted meat because of the current environmental situation.	
	CI6	I do believe that there are no negative consequences in consuming 3D-bioprinted meat.	
	CI7	I believe that people among society could benefit from the development and consumption of 3D-bioprinted meat.	
	CI8	I find 3D-bioprinted meat to be safe for consumption in the future.	
	CI9	I feel that developers put thorough processes in place and have developed 3D-bioprinted meat to make it safer to consume.	
Perceived Benefits	PB1	I seek healthier and safer meat options; thus, I will consider 3D-bioprinted meat.	[36]
	PB2	I prioritize food safety and reducing potential health hazards; thus, I will consider 3D-bioprinted meat.	
	PB3	I advocate for a more sustainable and responsible food system; thus, I will consider 3D-bioprinted meat.	
	PB4	I want to minimize animal suffering and promote a more humane approach to meat consumption; thus, I will consider 3D-bioprinted meat.	
	PB5	I envision a future of sustainable food production; thus, I will consider 3D-bioprinted meat.	
Perceived Barriers	BAR1	I seek to minimize contamination risks and ensure consistent quality of meat production; thus, I will consider 3D-bioprinted meat.	[36]
	BAR2	I am concerned about the environmental impact of traditional meat production; thus, I will consider 3D-bioprinted meat.	
	BAR3	I aspire to reduce animal suffering and eliminate the need for animal agriculture; thus, I will consider 3D-bioprinted meat.	
	BAR4	I am optimistic about tailoring meat products to individual preferences and dietary needs; thus, I will consider 3D-bioprinted meat.	
	BAR5	I want to embrace a transformative solution that addresses the limitations of traditional methods; thus, I will consider 3D-bioprinted meat.	
Health Concerns	HC1	I worry about getting sick from unknown diseases, but I see 3D-bioprinted meat as a safe and healthy alternative to traditional meat.	[66]
	HC2	I like that 3D-bioprinted meat is made in a clean and controlled environment, reducing the risk of contamination.	
	HC3	I am drawn to the safety and hygienic aspects of 3D-bioprinted meat production as the thought of becoming sick with an unknown infectious disease is unsettling.	
	HC4	I believe 3D-bioprinted meat can help protect my health and financial stability, considering the potential economic impact of falling ill.	
Self-Efficacy	SE1	I have the confidence and ability to learn about and utilize 3D-bioprinted meat as a new and innovative food option.	[65]
	SE2	I am encouraged to explore 3D-bioprinted meat if I see my family embrace the adoption of it.	
	SE3	I am confident that there are people and resources available to help me if I have any questions about 3D-bioprinted meat.	
	SE4	I am confident in my ability to navigate and access accurate and useful information about 3D-bioprinted meat online, ensuring I stay informed and make informed choices.	
	SE5	I believe I can confidently introduce 3D-bioprinted meat into my regular meals, adapting my cooking habits to this innovative food option.	
Altruistic Value	AV1	I believe that 3D-bioprinted meat has the potential to revolutionize the food industry and make healthy, sustainable protein accessible to everyone.	[45]
	AV2	I will support 3D-bioprinted meat because it helps address ethical issues in traditional meat production.	
	AV3	I believe 3D-bioprinted meat can make the world a more peaceful place by reducing the need to fight over food.	
	AV4	I will support 3D-bioprinted meat for its potential to provide a helpful, eco-friendly solution to environmental concerns, contributing to a more sustainable future.	
	AV5	I believe in the potential of 3D-bioprinted meat to foster a sense of shared responsibility for the well-being of our planet and its inhabitants.	
Egoistic Value	EV1	I will try 3D-bioprinted meat because it is the future of food.	[45]
	EV2	I will feel lucky to eat 3D-bioprinted meat because I can enjoy eating meat without harming animals or the environment.	
	EV3	I will consume 3D-bioprinted meat because it will make me part of a more sustainable and ethical food system.	
	EV4	I will consume 3D-bioprinted meat because I know that my choices influence others.	
	EV5	I will eat 3D-bioprinted meat because I am driven to make a positive impact on the world.	
Biospheric Value	BV1	I believe 3D-bioprinted meat can help Earth by reducing the need for animal agriculture.	[45]
	BV2	I believe 3D-bioprinted meat provides an eco-friendly and innovative approach to aligning our dietary choices with the environment.	
	BV3	I believe that 3D-bioprinted meat can reduce our impact on the planet by decreasing the need for animal agriculture, a major contributor to climate change, deforestation, and water pollution.	
	BV4	I believe that 3D-bioprinted meat can help reduce water and air pollution caused by animal agriculture.	
	BV5	I believe that supporting 3D-bioprinted meat reduces the ecological footprint associated with traditional meat production.	
Openness to Change Value	OC1	I am curious about the potential of 3D-bioprinted meat to revolutionize the way we enjoy food.	[45]
	OC2	I am thrilled about the endless possibilities of 3D-bioprinted meat to surprise and delight my taste buds.	
	OC3	I am eager to try 3D-bioprinted meat because it is a new food choice, fitting my openness to change.	
	OC4	I am attracted to the convenience of 3D-bioprinted meat.	
	OC5	I anticipate the benefits of 3D-bioprinted meat to be conversation-worthy to my family and friends.	
Ecological Worldview	EW1	I think that considering 3D-bioprinted meat is a way to support the natural world and be part of a community that cares about the environment.	[52]
	EW2	I feel disconnected from the natural world when I eat meat from animals that have been raised in factory farms.	
	EW3	I often feel connected with animals, and I believe that eating 3D-bioprinted meat is a way to honor their lives.	
	EW4	I believe that eating 3D-bioprinted meat is a way to show my respect for Earth and all of its inhabitants.	
	EW5	I believe that the welfare of the natural world is directly linked to my own well-being and eating 3D-bioprinted meat is a way to support the health of the planet.	
Awareness of Consequences	AC1	I am intrigued by the potential of 3D-bioprinted meat to reduce our reliance on finite resources.	[45]
	AC2	I am optimistic that 3D-bioprinted meat can improve quality of life.	
	AC3	I believe 3D-bioprinted meat can contribute to a cleaner and quieter atmosphere.	
	AC4	I am eager to embrace 3D-bioprinted meat as a way to reduce our environmental impact and promote respiratory health.	
	AC5	I am excited about the potential of 3D-bioprinted meat to create a more livable environment for all.	
Personal Norms	PN1	I feel personally obliged to choose 3D-bioprinted meat in an environmentally sound way.	[45]
	PN2	I believe I would be a better person if I choose 3D-bioprinted meat over traditional meat.	
	PN3	People like me should do whatever they can to minimize environmental issues, such as climate change caused by animal agriculture.	
	PN4	I feel guilty when I eat traditional meat while the environment is being damaged.	
	PN5	I feel a moral responsibility to prefer 3D-bioprinted meat whenever possible, regardless of what others do.	
Social Norms	SN1	If many people I respect and admire are already purchasing 3D-bioprinted meat, I will consider doing the same.	[64]
	SN2	If I notice a growing trend of people switching to 3D-bioprinted meat, I will be curious to learn more about this innovative product.	
	SN3	There is a growing expectation among my social circle to adopt sustainable food practices, and I feel a sense of responsibility to explore options like 3D-bioprinted meat.	
	SN4	The opinions of the people I value are shaping my perception of 3D-bioprinted meat, and I am open to trying this new product as a way to support their choices.	

**Table 2:** Demographical profile of the respondents (n = 738).

Characteristics	Category	n	%
Age	18–20 years old	95	12.87
	21–30 years old	241	32.66
	31–40 years old	181	24.53
	41–50 years old	192	26.02
	51 years old and above	29	3.93
Gender	Male	358	48.51
	Female	380	51.49
Educational Background	No schooling completed	6	0.81
	Elementary Graduate	7	0.95
	High School Graduate	360	48.78
	College Graduate	365	49.46
Occupation	Student	277	37.53
	Employed/Self-Employed	376	50.95
	Unemployed	68	9.21
	Retired	17	2.30
Frequency of traditional meat intake	Once or twice a week	82	11.11
	3–4 times a week	236	31.98
	5–6 times a week	170	23.04
	Daily	247	33.47
	None of the above	3	0.41

**Table 3:** Reliability and convergent validity result.

Construct	Items	Mean	S.D.	SWT (±1.96)	FL (≥0.7)	α (≥0.7)	CR (≥0.7)	AVE (≥0.5)
Consumption Intention	CI1	3.426	1.107	0.523	0.904	0.970	0.974	0.807
	CI2	3.345	1.129	0.438	0.889			
	CI3	3.372	1.104	0.540	0.911			
	CI4	3.399	1.057	0.723	0.910			
	CI5	3.498	1.087	0.708	0.908			
	CI6	3.064	1.204	0.168	0.816			
	CI7	3.456	1.061	0.698	0.919			
	CI8	3.298	1.084	0.401	0.912			
	CI9	3.490	1.061	0.843	0.911			
Perceived Benefits	PB1	3.401	1.094	0.563	0.915	0.956	0.966	0.850
	PB2	3.424	1.089	0.574	0.921			
	PB3	3.431	1.085	0.510	0.929			
	PB4	3.510	1.107	0.633	0.926			
	PB5	3.547	1.088	0.572	0.92			
Perceived Barriers	BAR1	3.488	1.061	0.819	0.927	0.959	0.968	0.859
	BAR2	3.470	1.086	0.562	0.923			
	BAR3	3.438	1.094	0.456	0.919			
	BAR4	3.458	1.068	0.552	0.942			
	BAR5	3.498	1.068	0.592	0.923			
Health Concerns	HC1	3.254	1.148	0.380	0.903	0.939	0.956	0.845
	HC2	3.475	1.070	0.804	0.922			
	HC3	3.411	1.067	0.655	0.926			
	HC4	3.264	1.104	0.372	0.926			
Self-Efficacy	SE1	3.485	1.066	0.592	0.925	0.948	0.960	0.829
	SE2	3.480	1.116	0.484	0.896			
	SE3	3.453	1.143	0.608	0.906			
	SE4	3.559	1.074	0.794	0.904			
	SE5	3.369	1.092	0.491	0.922			
Altruistic Value	AV1	3.369	1.083	0.502	0.894	0.940	0.954	0.806
	AV2	3.281	1.105	0.368	0.882			
	AV3	3.397	1.091	0.575	0.883			
	AV4	3.520	1.057	0.711	0.923			
	AV5	3.456	1.081	0.521	0.906			
Egoistic Value	EV1	3.298	1.091	0.456	0.879	0.944	0.957	0.817
	EV2	3.276	1.169	0.322	0.903			
	EV3	3.345	1.125	0.384	0.925			
	EV4	3.052	1.140	0.101	0.894			
	EV5	3.394	1.131	0.533	0.918			
Biospheric Value	BV1	3.591	1.117	0.430	0.919	0.950	0.961	0.832
	BV2	3.567	1.066	0.587	0.909			
	BV3	3.635	1.087	0.737	0.912			
	BV4	3.495	1.114	0.648	0.898			
	BV5	3.549	1.025	0.847	0.924			
Openness to Change Value	OC1	3.901	1.085	0.996	0.841	0.932	0.948	0.786
	OC2	3.530	1.113	0.490	0.899			
	OC3	3.458	1.146	0.436	0.907			
	OC4	3.328	1.144	0.341	0.892			
	OC5	3.392	1.108	0.479	0.892			
Ecological Worldview	EW1	3.498	1.094	0.452	0.874	0.928	0.946	0.778
	EW2	2.887	1.200	0.033	0.77			
	EW3	3.143	1.103	0.222	0.890			
	EW4	3.241	1.094	0.375	0.935			
	EW5	3.288	1.102	0.262	0.930			
Awareness of Consequences	AC1	3.663	1.058	0.781	0.873	0.945	0.958	0.819
	AC2	3.429	1.066	0.617	0.907			
	AC3	3.475	1.089	0.567	0.901			
	AC4	3.360	1.073	0.567	0.921			
	AC5	3.451	1.067	0.751	0.922			
Personal Norms	PN1	3.118	1.156	0.183	0.895	0.921	0.941	0.761
	PN2	2.901	1.177	0.121	0.888			
	PN3	3.601	1.080	0.748	0.787			
	PN4	2.973	1.196	0.022	0.862			
	PN5	3.047	1.170	0.162	0.924			
Social Norms	SN1	3.308	1.160	0.310	0.916	0.938	0.956	0.843
	SN2	3.520	1.118	0.692	0.915			
	SN3	3.239	1.166	0.372	0.911			
	SN4	3.342	1.148	0.579	0.93			

**Table 4:** Fornell–Larcker criterion result.

	AV	AC	BAR	PB	BV	CI	EW	EV	HC	OC	PN	SE	SN	Values
AV	0.898													
AC	0.857	0.905												
BAR	0.827	0.887	0.927											
PB	0.843	0.898	0.914	0.922										
BV	0.839	0.876	0.839	0.845	0.912									
CI	0.843	0.819	0.897	0.889	0.842	0.898								
EW	0.828	0.874	0.851	0.872	0.842	0.876	0.882							
EV	0.878	0.878	0.85	0.867	0.836	0.874	0.818	0.904						
HC	0.787	0.85	0.855	0.857	0.792	0.894	0.822	0.813	0.919					
OC	0.835	0.873	0.852	0.875	0.842	0.862	0.837	0.867	0.814	0.887				
PN	0.772	0.847	0.827	0.852	0.773	0.847	0.823	0.844	0.795	0.782	0.872			
SE	0.797	0.857	0.867	0.856	0.806	0.886	0.819	0.822	0.852	0.847	0.791	0.911		
SN	0.744	0.818	0.818	0.821	0.748	0.838	0.814	0.803	0.801	0.808	0.801	0.839	0.918	
Values	0.844	0.824	0.756	0.791	0.733	0.712	0.803	0.835	0.847	0.833	0.845	0.823	0.823	0.851

**Table 5:** Heterotrait–Monotrait (HTMT) ratio.

	AV	AC	BAR	PB	BV	CI	EW	EV	HC	OC	PN	SE	SN	Values
AV														
AC	0.809													
BAR	0.771	0.832												
PB	0.789	0.845	0.754											
BV	0.787	0.825	0.779	0.787										
CI	0.782	0.838	0.829	0.834	0.775									
EW	0.779	0.824	0.795	0.819	0.788	0.818								
EV	0.831	0.829	0.792	0.812	0.781	0.813	0.836							
HC	0.736	0.802	0.8	0.803	0.737	0.836	0.777	0.763						
OC	0.791	0.832	0.801	0.827	0.795	0.805	0.791	0.823	0.768					
PN	0.729	0.806	0.779	0.808	0.727	0.796	0.762	0.804	0.754	0.742				
SE	0.742	0.805	0.808	0.797	0.747	0.821	0.765	0.766	0.801	0.8	0.744			
SN	0.692	0.768	0.762	0.767	0.692	0.778	0.768	0.753	0.753	0.764	0.76	0.789		
Values	0.785	0.786	0.822	0.84	0.768	0.831	0.839	0.792	0.787	0.817	0.789	0.797	0.758	

**Table 6:** Model fit result.

Model Fit	Parameter Estimates	Minimum Cutoff	Recommended by
SRMR	0.054	<0.08	Hu and Bentler [77]
Chi-square/dF	3.217	<5.00	Hooper et al. [78]
NFI	0.943	>0.90	Baumgartner and Homburg [79]

**Table 7:** Respondents’ hypothesis testing.

Hypothesis	Relationship	Beta	*p*-Value	Significance	Decision
1	PB → CI	0.136	0.005	Significant	Accept
2	BAR → CI	0.129	0.009	Significant	Accept
3	HC → CI	0.253	<0.001	Significant	Accept
4	SE → CI	0.166	<0.001	Significant	Accept
5	Values → EW	0.903	<0.001	Significant	Accept
6	EW → AC	0.874	<0.001	Significant	Accept
7	AC → PN	0.582	<0.001	Significant	Accept
8	AC → CI	0.175	<0.001	Significant	Accept
9	PN → CI	0.097	<0.001	Significant	Accept
10	SN → PN	0.325	<0.001	Significant	Accept
11	SN → CI	0.058	0.047	Significant	Accept
HO	Values → AV	0.944	<0.001	Significant	Accept
HO	Values → BV	0.933	<0.001	Significant	Accept
HO	Values → EV	0.954	<0.001	Significant	Accept
HO	Values → OC	0.933	<0.001	Significant	Accept

## Data Availability

The original contributions presented in the study are included in the article, further inquiries can be directed to the corresponding author.
